# Negation of Belief Function Based on the Total Uncertainty Measure

**DOI:** 10.3390/e21010073

**Published:** 2019-01-15

**Authors:** Kangyang Xie, Fuyuan Xiao

**Affiliations:** School of Computer and Information Science, Southwest University, No. 2 Tiansheng Road, BeiBei District, Chongqing 400715, China

**Keywords:** Dempster-Shafer theory, negation, total uncertainty measure, basic probability assignment

## Abstract

The negation of probability provides a new way of looking at information representation. However, the negation of basic probability assignment (BPA) is still an open issue. To address this issue, a novel negation method of basic probability assignment based on total uncertainty measure is proposed in this paper. The uncertainty of non-singleton elements in the power set is taken into account. Compared with the negation method of a probability distribution, the proposed negation method of BPA differs becausethe BPA of a certain element is reassigned to the other elements in the power set where the weight of reassignment is proportional to the cardinality of intersection of the element and each remaining element in the power set. Notably, the proposed negation method of BPA reduces to the negation of probability distribution as BPA reduces to classical probability. Furthermore, it is proved mathematically that our proposed negation method of BPA is indeed based on the maximum uncertainty.

## 1. Introduction

Information representation has been a crucial issue since the emergence of artificial and intelligent systems [1,2]. Human beings are able to recognize and transform the world by obtaining and recognizing information in nature and society to distinguish different things [3]. However, representing uncertain information from different information sources is still an open issue [4,5]. A large amount of literature and a number of approaches have been proposed to settle this issue, such as Dempster-Shafer theory (DST) [6,7,8,9,10], fuzzy sets theory [11,12,13,14,15], entropy [16,17,18,19,20], D-number theory [21,22,23,24] and Z-numbers [25].

In some particular circumstances, it is likely easier to say what it is not than to say what it is, since more information may be needed to describe what it is while less information may be needed to describe what it is not. For example, sometimes it is difficult to prove whether a theorem is correct or incorrect by mathematical approaches directly; however, just a particular counterexample can easily prove a statement wrong. A more intuitive example is that it must be easier to obtain a probability of a complex event by using the unit one subtracts from the probability of a simple event that is exactly the complement of the complex event, rather than calculating the probability of the complex event directly. Therefore, in this paper we try to solve the issue from the opposite side, that is to find out what is the negation of it [26].

Increasing attention has been paid to negation since it was raised by Zadeh. It is of great significance to study negation since it enables us to obtain information from the opposite side and also represent the information through the opposite side [27]. Furthermore, the measure of fuzziness proposed by Yager suggested that fuzziness can be related to the lack of distinction between a set and its negation [28]. That is, the less distinct A and $\bar{A}$ the more fuzzy A. Moreover, the negation method is also promising in muti-criteria decision (MCDM) making. For example, one of the most used methods is TOPSIS (Technique for Order Preference by Similarity to an Ideal Solution), which provides us with an ideal solution (IS) and the negative ideal solution (NIS). The best alternative is as close to the ideal solution and is as far from the negative ideal solution as possible. As a result, taking the negation side is meaningful in MCDM. In addition, the negation of BPA can also be applied in measuring the uncertainty of BPA [29]. Thus, obtaining the negation is of great significance. Recently, a negation method of a probability distribution based on the maximum entropy has been proposed and studied [27]. Some properties have been investigated regarding Yager’s negation [30]. The negation of a probability distribution can be seen as a reallocation process of probability value. In this paper, we try to extend the negation method of a probability distribution in classical probability theory to a basic probability assignment in D-S theory, which provides a novel view of the expression of uncertainty and unknown in D-S theory. A novel negation method of basic probability assignment (BPA) in Dempster-Shafer theory is proposed in a matrix form as well as the BPA which is analogous to the fact that a probability distribution can be represented as a vector. To study the evolution of a BPA vector in the repeated negation process, a total uncertainty measure $H_{b}\left(m\right)$ proposed by Pal et al. [31,32] is adopted in this paper to measure the uncertainty of basic probability assignment (BPA). Properties of the proposed negation method are presented and proved. Compared with the negation method of a probability distribution, the proposed negation method of BPA differs by the fact that the BPA of a certain element is reassigned to the other elements in the power set where the weight of reassignment is proportional to the cardinality of intersection of the element and each remaining element in the power set. Notably, the proposed negation method of BPA reduces to the negation of probability distribution as BPA reduces to classical probability.

The rest of this paper is structured as follows: Some basic knowledge associated with D-S theory and uncertainty measurement are presented in Section 2. In Section 3, the negation method for BPA is proposed, some numerical examples are presented and some properties are discussed and proved. Finally, findings are summarized in Section 4.

## 2. Preliminaries

### 2.1. Dempster-Shafer Theory

Dempster-Shafer theory (DST) was first proposed by Dempster [33] in 1967 and refined by his student Shafer [34] in 1976. It has been widely applied to decision-making [35,36,37,38,39,40,41,42], information fusion [43], fault diagnosis [44,45], uncertain reasoning [46,47,48], multi-sensor data fusion [49,50,51], aggregation [52,53], medical diagnosis [54], conflicting management [55] and other fields [56,57,58,59] owing to its ability to express uncertainty.

Let Θ be a set of mutually exclusive and exhaustive hypothesis called the frame of discernment (FOD) which has *N* elements and is indicated as:

(1) $$\Theta =\{H_{1},H_{2},H_{3},\cdots ,H_{n}\}$$

The power set of Θ consists of all subsets of Θ, containing 2${}^{N}$ elements is indicated as [33,34]:

(2) $$2^{\Theta }=\left\{\emptyset ,\left\{H_{1}\right\},\left\{H_{2}\right\},\cdots ,\left\{H_{n}\right\},\left\{H_{1},H_{2}\right\},\cdots ,\theta \right\}$$

where ∅ denotes the empty set and θ denotes the whole set. A crucial concept in D-S theory is Basic Probability Assignment (BPA), the mass of belief in an element of Θ is analogous to a probability distribution, but differs by the fact that the unit mass is distributed among 2${}^{\Theta }$ elements instead of *N* elements, which means the mass of belief is assigned to not only singletons but also composite hypothesises. The mass function is a mapping from 2${}^{\Theta }$ to [0-1] representing how strongly the evidence supports the hypothesis indicated as [33,34]:

(3) $$2^{\Theta }\to \left[0,1\right]$$

(4) m(∅)=0

(5) $$\sum_{A\subseteq \Theta }m\left(A\right)=1$$

*A* is named as a focal element of *m* (mass function) if *A* $\subset 2^{\Theta }$ and m(A) ≠ 0. Basic probability assignment reduces to basic probability distribution when all focal elements reduce to singletons.

According to the Basic Probability Assignment (BPA), the plausibility function $PL_{m}\left(A\right)$ and belief function $Bel_{m}\left(A\right)$ are defined as:

(6) $$PL_{m}\left(A\right)=\sum_{B\in 2^{\Theta },B\cap A\neq \emptyset }m\left(B\right)$$

(7) $$Bel_{m}\left(A\right)=\sum_{B\in 2^{\Theta },B\subseteq A}m\left(B\right)$$

The plausibility function $PL_{m}\left(A\right)$ measures the potential belief to *A*, which means the total belief that does not negate *A*, while the belief function $Bel_{m}\left(A\right)$ measures total belief to *A*.

### 2.2. Uncertainty Measurements of Basic Probability Assignment (BPA)

Measuring uncertainty has been a key problem in information science [60,61,62]. The concept of entropy is derived from physics, which has been a measure of uncertainty and disorder [63]. Generally, a system with higher uncertainty has greater entropy, which also contains more information [64,65]. Shannon entropy is widely adopted to measure the uncertainty of a probability distribution [66], which is defined as [67]:

(8) $$H\left(P\right)=-\sum_{i=1}^{n}P_{i}log_{b}P_{i}$$

where *n* is the total number of all events in an experiment, $P_{i}$ is the probability that the ith event happens meeting $\sum_{i}^{n}P_{i}=1$. Generally, 2 is adopted as the base of logarithm, and the unit of entropy is bit. Shannon entropy hits the maximum when the unit is assigned to each event equally, which also hits the maximum uncertainty.

However, the measurement of uncertainty is still an open issue in D-S theory. Heterogeneous definitions and requirements of uncertainty measure [68,69,70,71,72,73] are proposed to measure the uncertainty in D-S theory.

A total uncertainty measure $H_{b}\left(m\right)$ proposed by Pal et al. [31,32] is adopted in this paper to measure the uncertainty of probability assignment (BPA), which is defined as [32]:

(9) $$H_{b}\left(m\right)=\sum_{A\in 2^{\Theta }}m\left(A\right)\log_{2}\left(\frac{\left|A\right|}{m(A)}\right)$$

where $\left|A\right|$ denotes the cardinality of *A*, meaning the total number of elements in *A*. $H_{b}\left(m\right)$ has many advantages, such as consistency with D-S theory semantics, monotonicity, probability consistency and additivity properties [70]. The total uncertainty measure has a unique maximum for m such that $m\left(A\right)\propto \left|A\right|$ is satisfied [32], where ∝ denotes ‘be proportional to’. It is noted that the total uncertainty measure reduces to basic Shannon entropy when all focal elements in D-S theory reduce to singletons in classical probability theory.

A new definition of entropy of belief functions is defined as [70]:

(10) $$H_{rp}\left(m\right)=\sum_{\theta \in \Theta }Pl\_P_{m}\left(\theta \right)log\left(\frac{1}{Pl\_P_{m}\left(\theta \right)}\right)+\sum_{A\in 2^{\Theta };A\neq \emptyset }m\left(A\right)log\left(|A|\right)$$

where

(11) $$Pl\_P_{m}\left(\theta \right)=K^{-1}Pl_{m}\left(\theta \right),K=\sum_{\theta \in \Theta }Pl_{m}\left(\theta \right)$$

Deng entropy is defined as [69]:

(12) $$H_{d}\left(m\right)=\sum_{A\subseteq 2^{\Theta }}m\left(A\right)log\left(\frac{2^{\left|A\right|}-1}{m(A)}\right)$$

### 2.3. Negation of Probability Distribution

Information that is contained in the negation is hardly considered in information representation. To solve this problem, Yager proposed a negation method of probability distribution, which is concerned with the information representation contained in the negation of a probability distribution [27]. Considering a probability distribution

(13) $$P=(p_{1},p_{2},p_{3},\cdots ,p_{n})$$

defined on the set $X=(x_{1},x_{2},x_{3},\cdots ,x_{n})$ where $0\leq p_{i}\leq 1$ and $\sum_{i=1}^{n}p_{i}=1$. The negation of the probability distribution is denoted by ${\bar{p}}_{i}$ and defined as [27]:

(14) $$\bar{P}=\left({\bar{p}}_{1},{\bar{p}}_{2},{\bar{p}}_{3},\cdots ,{\bar{p}}_{n}\right)$$

where

(15) $${\bar{p}}_{i}=\frac{1-p_{i}}{n-1}$$

According to Equation (10), each negation operation could be regarded as a process to reassign the probability value $p_{i}$ among the n−1 other hypothesises equally. Namely [27]:

(16) $${\bar{p}}_{i}=\frac{\sum_{j=1,i\neq j}^{n}p_{j}}{n-1}$$

The negation operation can also be interpreted on a different view if we observe that [27]:

(17) $${\bar{p}}_{i}=\frac{1-p_{i}}{\sum_{i=1}^{n}\left(1-p_{i}\right)}$$

${\bar{p}}_{i}$ is obtained by normalizing the complementary of $p_{i}$ (a probability value) to make sure the sum equals 1. Furthermore, the repeated process of negation can be modeled as a difference equation denoted as [27]:

(18) $$\left(n-1\right)p_{i}\left(k+1\right)+p_{i}\left(k\right)=1$$

The solution of this difference equation approaches 1/n as *i* increases, which means the unit probability value is equally allocated to each element in *X*. If we back to review the definition of Shannon entropy then, it is not hard to find that the maximum value of Shannon entropy is attained exactly for this uniform distribution, which demonstrates that the maximum value of uncertainty of the system is attained. Moreover, it is proved that the Shannon entropy increases constantly as the iteration of negation process increases.

According to the analysis of negation of probability distribution, three important properties are summarized as follows:

1. Repeated process of negation of probability distribution converges to a certain probability distribution.
2. The maximum value of uncertainty of the system is calculated exactly for the convergent probability distribution.
3. The entropy increases constantly till the maximum value of the total uncertainty attains.

We apply these three properties of negation to D-S theory and define a negation method of BPA in the following section.

## 3. Negation of BPA

### 3.1. Definition of Negation

D-S theory has been widely used in expressing information [74] and other fields [51,60,75] since the ability to deal with uncertainty and unknown with weaker conditions than Bayesian probability theory. In this paper, a novel negation method of BPA is proposed.

Consider a frame of discernment Θ containing *N* elements, then the power set of Θ containing $2^{N}$ elements is denoted as:

(19) $$2^{\Theta }=\left\{H_{1},H_{2},H_{3},\cdots ,H_{2^{N}}\right\}$$

where $H_{1}$ denotes ∅ and $H_{2^{N}}$ denotes θ. Let *m* be a BPA, which is represented in vector form:

(20) $$\vec{m}={\left[m_{1},m_{2},m_{3},\cdots ,m_{2^{N}}\right]}^{T}$$

assuming that

(21) $$m_{i}=m\left(H_{i}\right)$$

where

(22) $$\sum_{i=1}^{2^{n}}m_{i}=1,m_{i}\in \left[0,1\right]$$

similarly, the vector form of the negation of BPA is defined as:

(23) $$\vec{\bar{m}}={\left[{\bar{m}}_{1},{\bar{m}}_{2},{\bar{m}}_{3},\cdots ,{\bar{m}}_{2^{N}}\right]}^{T}$$

Given a BPA vector $\vec{m}$, the negation of the BPA is defined as:

(24) $$\vec{\bar{m}}=E\vec{m}$$

where *E* is the negation matrix defined as:

(25) $$E=\left[\begin{array}{cccc} e_{1,1} & e_{1,2} & \cdots & e_{1,2^{N}}\\ e_{2,1} & e_{2,2} & \cdots & e_{2,2^{N}}\\ \vdots & \vdots & \ddots & \vdots \\ e_{2^{N},1} & e_{2^{N},2} & \cdots & e_{2^{N},2^{N}} \end{array}\right]$$

When $j\neq 2^{N}$ and j≠1:

(26) $$e_{i,j}=\left\{\begin{array}{cc} 0 & i=j\\ \frac{\left|H_{i}\cap \bar{H_{j}}\right|}{\sum_{H_{k}\neq \theta ,H_{k}\neq \emptyset ,H_{k}\in 2^{\Theta }}\left|H_{k}\cap \bar{H_{j}}\right|} & i\neq j \end{array}\right.$$

where ${\bar{A}}_{j}=\Theta -A_{j}$, $|H_{i}|$ denotes the cardinality of $H_{i}$.

When $j=2^{N}$, as $A_{j}=\theta$:

(27) $$e_{i,j}=\left\{\begin{array}{cc} 1 & i=j\\ 0 & i\neq j \end{array}\right.$$

when j=1, as $A_{j}$ = ∅:

(28) $$e_{i,j}=0$$

when $i=2^{N}$:

(29) $$e_{i,j}=\left\{\begin{array}{cc} 1 & i=j\\ 0 & i\neq j \end{array}\right.$$

### 3.2. Steps of Constructing the Negation

In classical probability theory, the negation of a probability distribution *P* is obtained by allocating its probability $p_{i}$ equally among the n−1 other elements [27]. Similarly, for each $H_{i}$ in $2^{\Theta }$ the negation of BPA is constructed by reassigning its mass $m_{i}$ to those elements $\in 2^{\Theta }$ whose intersection with complement of $H_{i}$ is not empty set. Specifically, the BPA in $H_{i}$($H_{i}\in 2^{\Theta }$) is reassigned to other elements in the power set without $H_{i}$. Furthermore, the negation of BPA is distinct from the negation of a probability distribution by the fact that the reassignment weight of BPA is proportional to the cardinality of intersection of the element and each remaining element in the power set. For example, consider that the frame of discernment is {a,b,c} then {a} allocates twice BPA to $\left\{\bar{bc}\right\}$ than $\left\{\bar{ac}\right\}$, since the cardinality of intersection of {bc} (complement of {a}) and {bc} is 2, while the cardinality of intersection of {bc} (complement of {a}) and {ac} is 1. Therefore, we are concerned with not only the belief degree of the focal elements but also the cardinality of the intersection that can affect the negation of BPA. Thus, the procedure of obtaining an element $e_{i,j}$ in the negation matrix could be described in three steps:

**Step 1:** Obtain the element in the $\bar{A_{j}}$ by

(30) $$\bar{A_{j}}=\Theta -A_{j}$$

since $\Theta -A_{j}$ represents the complementary elements of $A_{j}$, where Θ denotes the frame of discernment (FOD).

**Step 2:** Calculate the cardinality of intersection of $A_{i}$ and $\bar{A_{j}}$, which is the reallocation weight of negation process for $H_{j}$ denoted as $c_{j}$ and the sum σ of the cardinality from j=2 to $j=2^{N}-1$ (except for empty set and the whole set). Namely:

(31) $$\sigma =\sum_{j=2}^{2^{N}-1}c_{j}$$

**Step 3:** Normalize these weights of negation process of $H_{j}$ to guarantee their sum is one.

(32) $$e_{i,j}=\frac{c_{j}}{\sigma }$$

Consequently, the general formula of elements in negation matrix is denoted as

(33) $$e_{i,j}=\frac{|A_{i}\cap \bar{A_{j}}|}{\sum_{A_{k}\neq \Theta ,A_{k}\in 2^{\Theta }}\left|A_{k}\cap \bar{A_{j}}\right|}$$

It is noted that $\bar{m_{i}}$ is BPA since each focal element allocates its BPA to some other focal elements in the power set and the BPA in the whole set retains, which gives that

(1) $\sum_{i=1}^{i=2}{\bar{m}}_{i}=1$
(2) ${\bar{m}}_{i}\in \left[0,1\right]$

According to the definition of the negation of BPA, essentially, the negation of BPA is a process of reassignment of BPA in a particular manner. It could be noted that the ith column in the negation matrix represents the allocation weight of $p_{i}$ and the *j*th row in the negation matrix tells us the allocation weight of the given BPA vector.

First of all, the negation of two special elements in the power set (∅ and θ, which means empty set and the whole set, respectively) are discussed.

We assume that the frame of discernment is exhaustive (close-world assumption, proposed by Yager [76]), which means information sources are reliable. Thus, according to the close-world assumption, BPA of empty set (∅) is always 0, no matter how many times negation process is applied. Thus, to make sure the BPA of the empty set is always 0, elements in the first column and the first row of the negation matrix are all defined as 0, which means the other focal elements cannot allocate their BPA to the empty set when the negation process is applied.

In D-S theory, it should be noted that the BPA of the whole set (θ) denotes the belief of total uncertainty that it has no idea where to allocate the BPA in the whole set. Furthermore, compared with the whole set, the $2^{N}-2$ other (except for empty set) elements are relatively certain and definitely know what elements are not in the hypothesis, which means the complement of them is not the empty set ∅. In this case, since the whole set represents total uncertainty that does not know where to allocate the BPA belonging to the whole set, Similarly, it does not know where to allocate its BPA when a negation process is constructed either. Thus, we define that the last column of the negation matrix is 0 except for the last element to make sure that the BPA of the whole set cannot be allocated to the $2^{N}-1$ other focal elements when negation process is applied. Furthermore, according to the close-world assumption which means the frame of discernment is complete and exhaustive, each complement of focal element except for the whole set is relatively certain, and so is the negation of these focal elements, which cannot be totally uncertain (θ). Thus, the BPA of $2^{N}-1$ focal elements (except for the whole set θ) cannot be allocated to the whole set when negation process is applied. In this case, we again define that the last row of the negation matrix is 0 except for the last element to guarantee the $2^{N}-1$ other elements are unable to allocate their BPA to the whole set when negation process is applied. Therefore, the whole set is unable to allocate its BPA to any other focal elements while any other focal elements cannot allocate their BPA to the whole set when negation process is applied. Therefore, the BPA in the whole set remains constant when negation process is applied.

### 3.3. Numerical Examples of the Negation Process

**Example** **1.** *Assume that the frame of the discernment has only one element Θ={a}, then of course we have m(a)=1 according to the definition of uncertainty measurement above, the total uncertainty is calculated as:*

(34) $$H_{b}\left(m\right)=0$$

*and the negation of the BPA is calculated as:*

(35) $$\vec{\bar{m}}=E\vec{m}$$

*to be more specific:*

(36) $$\left[\begin{array}{c} \emptyset \\ \bar{m}\left(a\right) \end{array}\right]=\left[\begin{array}{cc} 0 & 0\\ 0 & 1 \end{array}\right]\left[\begin{array}{c} \emptyset \\ m(a) \end{array}\right]$$

*it follows from Equation (36) that:*

(37) $$\bar{m}\left(a\right)=m\left(a\right)$$

*Furthermore, the total uncertainty of $\bar{m}$ is calculated as:*

(38) $$H_{b}\left(m\right)=0$$

*Since N=1, the singleton {a} is regarded as the whole set {Θ}. Thus, the BPA remains constant after the negation process. In this case, no matter how many times the negation process is applied, the BPA remains unchanged and so does the total uncertainty.*

**Example** **2.** *The special case is noted for N=2. Assume a frame of discernment Θ={a,b}, for a BPA vector*

(39) $$\vec{m}=\left[\begin{array}{c} \emptyset \\ m(a)\\ m(b)\\ m(ab) \end{array}\right]=\left[\begin{array}{c} 0\\ 0.2\\ 0.3\\ 0.5 \end{array}\right]$$

*According to Equation (7), the total uncertainty of the original BPA is:*

(40) $$H_{b}\left(m\right)=1.9855$$

*it follows from Equations (20)–(24) that the negation matrix is obtained as:*

(41) $$E=\left[\begin{array}{cccc} 0 & 0 & 0 & 0\\ 0 & 0 & 1 & 0\\ 0 & 1 & 0 & 0\\ 0 & 0 & 0 & 1 \end{array}\right]$$

*Since the BPA of the whole set ({ab}) retains unchanged, the BPA of m(a) reassigns to $\bar{m}\left(b\right)$ and the BPA of m(b) reassigns to $\bar{m}\left(a\right)$, which means*

(42) $$\bar{m}\left(a\right)=m\left(b\right)$$

(43) $$\bar{m}\left(b\right)=m\left(a\right)$$

*which means*

(44) $$\vec{\bar{m}}=\left[\begin{array}{c} 0\\ 0.3\\ 0.2\\ 0.5 \end{array}\right]$$

*The total uncertainty of $\bar{m}$ is*

(45) $$H_{b}\left(\bar{m}\right)=1.9855$$

*Clearly, for N=2, the uncertainty of the system retains unchanged, no matter how many times the negation process is applied. This property is consistent with order reversal of the negation of probability distribution proposed by Yager, and for the special case N=2, the negation of BPA is consistent with the negation of probability distribution [27].*

**Example** **3.** *For a more general case, assume a frame of discernment consists of three elements Θ={a,b,c} for a BPA vector*

(46) $$\vec{m}=\left[\begin{array}{c} \emptyset \\ m(a)\\ m(b)\\ m(c)\\ m(ab)\\ m(ac)\\ m(bc)\\ m(abc) \end{array}\right]=\left[\begin{array}{c} 0\\ 0.1\\ 0.15\\ 0\\ 0\\ 0.3\\ 0.25\\ 0.2 \end{array}\right]$$

*The total uncertainty measure of m is*

(47) $$H_{b}\left(m\right)=3.0952$$

*According to the definition of proposed negation method, the negation matrix is derived as:*

(48) $$E=\left[\begin{array}{cccccccc} 0 & 0 & 0 & 0 & 0 & 0 & 0 & 0\\ 0 & 0 & \frac{1}{6} & \frac{1}{6} & 0 & 0 & \frac{1}{3} & 0\\ 0 & \frac{1}{6} & 0 & \frac{1}{6} & 0 & \frac{1}{3} & 0 & 0\\ 0 & \frac{1}{6} & \frac{1}{6} & 0 & \frac{1}{3} & 0 & 0 & 0\\ 0 & \frac{1}{6} & \frac{1}{6} & \frac{1}{3} & 0 & \frac{1}{3} & \frac{1}{3} & 0\\ 0 & \frac{1}{6} & \frac{1}{3} & \frac{1}{6} & \frac{1}{3} & 0 & \frac{1}{3} & 0\\ 0 & \frac{1}{3} & \frac{1}{6} & \frac{1}{6} & \frac{1}{3} & \frac{1}{3} & 0 & 0\\ 0 & 0 & 0 & 0 & 0 & 0 & 0 & 1 \end{array}\right]$$

*the negation of $\vec{m}$ is calculated as:*

(49) $$\vec{\bar{m}}=E\vec{m}=\left[\begin{array}{c} \emptyset \\ \vec{m}\left(a\right)\\ \vec{m}\left(b\right)\\ \vec{m}\left(c\right)\\ \vec{m}\left(ab\right)\\ \vec{m}\left(ac\right)\\ \vec{m}\left(bc\right)\\ \vec{m}\left(abc\right) \end{array}\right]=\left[\begin{array}{c} 0\\ 0.1083\\ 0.1167\\ 0.0417\\ 0.2250\\ 0.1500\\ 0.1583\\ 0.2000 \end{array}\right]$$

*The total uncertainty measure of $\bar{m}$ is:*

(50) $$H_{b}\left(\bar{m}\right)=3.5305$$

*Repeat the negation process once again $\vec{\bar{\bar{m}}}$ is obtained as:*

(51) $$\vec{\bar{\bar{m}}}=\left[\begin{array}{c} \emptyset \\ \bar{\vec{m}}\left(a\right)\\ \bar{\vec{m}}\left(b\right)\\ \bar{\vec{m}}\left(c\right)\\ \bar{\vec{m}}\left(ab\right)\\ \bar{\vec{m}}\left(ac\right)\\ \bar{\vec{m}}\left(bc\right)\\ \bar{\vec{m}}\left(abc\right) \end{array}\right]=\left[\begin{array}{c} 0\\ 0.07921\\ 0.0750\\ 0.1125\\ 0.1542\\ 0.1917\\ 0.1875\\ 0.2000 \end{array}\right]$$

*The total uncertainty measure of $\vec{\bar{\bar{m}}}$ is*

(52) $$H_{b}\left(\bar{\bar{m}}\right)=3.5649$$

**Figure 1:**
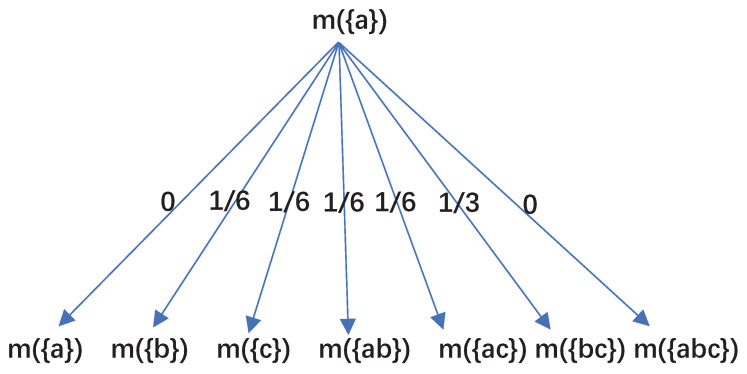
Reallocation weight of m(a).

It is noted that the BPA of m(a) is reassigned to {b},{c},{ab},{ac} and {bc} with the proportion of 1:1:1:1:2. However the BPA of the whole set remains unchanged after the negation process is applied. Specifically, Figure 1 illustrates the weight of reallocation of m(a) for N=3 intuitively.

### 3.4. Discussion

Recall the general case of Example 3, applying 15 successive negation process to the BPA vector in Example 3, and the results are shown in Table 1.

**Figure 2:**
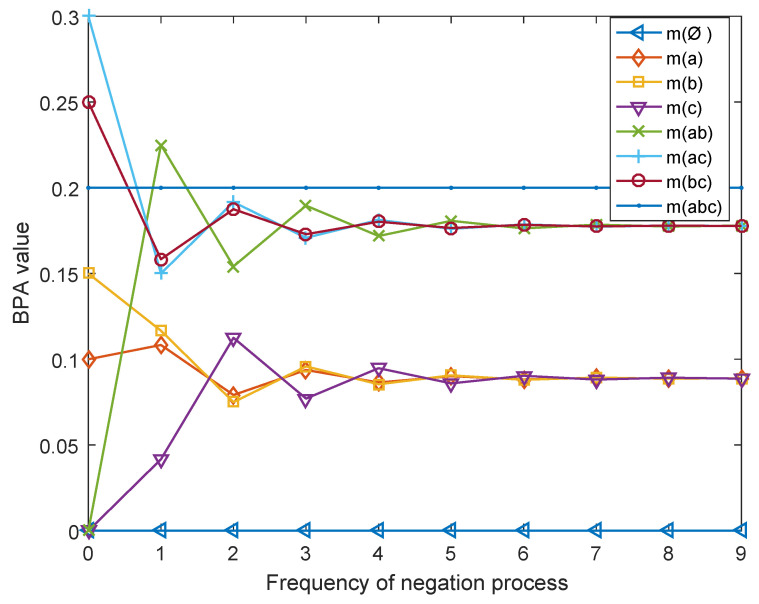
Evolution of basic probability assignment (BPA) as iteration of negation process increases.

It is noted that the BPA of each focal element converges to the proportion by degrees that m(a):m(b):m(c):m(ab):m(ac):m(bc) = 1:1:1:2:2:2 and the total uncertainty increases constantly till it attains 3.5749, which is the maximal value of the total uncertainty for N=3, as the iteration of negation process increases. To be more intuitive, the evolution of BPA as the iteration of negation process increases is illustrated in Figure 2.

**Figure 3:**
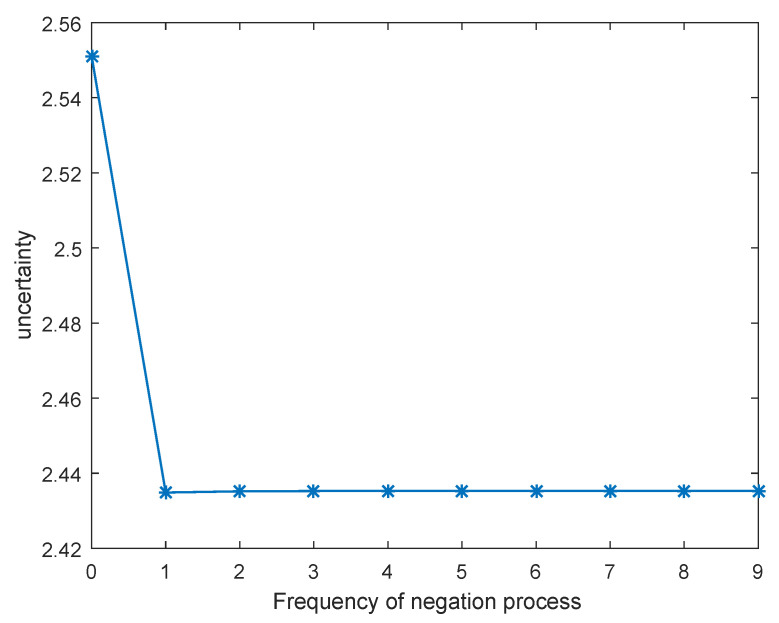
Uncertainty measured by $H_{rp}\left(m\right)$ as iteration of negation process increases.

**Figure 4:**
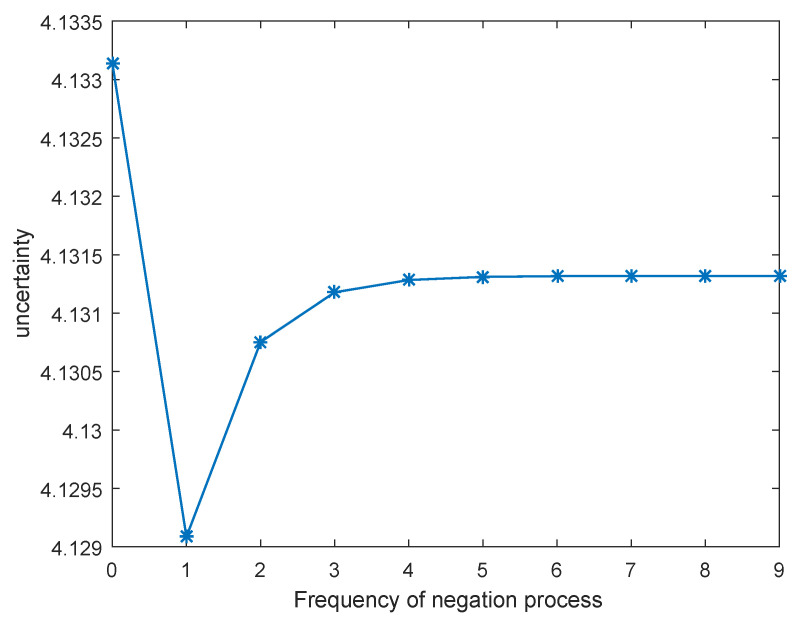
Uncertainty measured by $H_{d}\left(m\right)$ as iteration of negation process increases.

Recall that $\bar{\bar{m}}\neq \bar{m}$ (except for N=1 and N=2), according to Figure 2 and Table 1, this phenomenon could result from the fact that the total uncertainty measure increases after each negation process, which means that the uncertainty of the system increases after each negation process. To be more specific, the uncertainty of the BPA of m(a) = 0.05, m(b) = 0.05, m(c) = 0.05, m(ab) = 0.2, m(ac) = 0.2, m(bc) = 0.25, m(abc) = 0.2 measured by the two other uncertainty measures are compared in Table 2. It is illustrated in Table 2 that the uncertainty only increases constantly in the total uncertainty measure, while the uncertainty measured by the two other uncertainty measures fluctuate back and forth. The change of uncertainty measured by $H_{rp}\left(m\right)$ and $H_{d}\left(m\right)$ is showed in Figure 3 and Figure 4, respectively. The uncertainty is unable to increase constantly when measured by $H_{rp}\left(m\right)$ and $H_{d}\left(m\right)$, which is against our proposed negation method based on the maximum uncertainty. Therefore, the total uncertainty measure is mainly discussed in this Section.

Since we are trying to extend the negation of a probability distribution to a belief function, we argue that some particular properties of the negation of a mass function should be compatible and consistent with the negation of a probability distribution, proposed by Yager [27]. According to Yager’s idea ‘the reason that one selects maximum entropy alternatives is that it picks the allowable alternative which brings with it the least unsupported information’ and it is proved that the entropy increases once after a negation process [27]. Therefore, it is necessary that the uncertainty should increase constantly as the iteration of the negation process, in order to be compatible and consistent with the negation of a probability distribution.

According to Equation (7) the total uncertainty of $\vec{\bar{m}}$ can be measured as:

(53) $$H_{b}\left(\bar{m}\right)=\sum_{A\in 2^{\Theta }}\bar{m}\left(A\right)log_{2}\left(\frac{\left|A\right|}{\bar{m}\left(A\right)}\right)$$

Thus, the increase in the total uncertainty obtained by the negation process is denoted as the difference between the two uncertainties:

(54) $$H_{b}\left(\bar{m}\right)-H_{b}\left(m\right)$$

Since the empty set ∅ and the whole set θ have no effect on the difference between the two uncertainties it can be simplified as:

(55) $$\begin{array}{c} H_{b}\left(\bar{m}\right)-H_{b}\left(m\right)=\\ \sum_{A\in 2^{\Theta },A\neq \emptyset ,A\neq \Theta }\bar{m}\left(A\right)log_{2}\left(\frac{\left|A\right|}{\bar{m}\left(A\right)}\right)-\\ \sum_{A\in 2^{\Theta },A\neq \emptyset ,A\neq \Theta }m\left(A\right)log_{2}\left(\frac{\left|A\right|}{m(A)}\right) \end{array}$$

To avoid redundant descriptions, empty set ∅ and the whole set θ will not be considered in calculation of two entropies $H_{b}\left(m\right)$ and $H_{b}\left(\left(\bar{m}\right)\right)$ in proving $H_{b}\left(\bar{m}\right)\geq H_{b}\left(m\right)$.

Consider a frame of discernment Θ={a,b,c} then according to the negation matrix in Equation (43) each element in $\vec{\bar{m}}$ can be denoted by elements in $\vec{m}$ as Table 3:

Thus the total uncertainty of $H_{b}\left(\bar{m}\right)$ can be denoted as:

(56) $$\begin{array}{c} \log_{2}({\left(\frac{1}{\bar{m}\left(a\right)}\right)}^{\bar{m}\left(a\right)}\cdot {\left(\frac{1}{\bar{m}\left(b\right)}\right)}^{\bar{m}\left(b\right)}\cdot {\left(\frac{1}{\bar{m}\left(c\right)}\right)}^{\bar{m}\left(c\right)}\cdot \\ {\left(\frac{2}{\bar{m}\left(ab\right)}\right)}^{\frac{1}{2}\bar{m}\left(ab\right)}\cdot {\left(\frac{2}{\bar{m}\left(ab\right)}\right)}^{\frac{1}{2}\bar{m}\left(ab\right)}\cdot {\left(\frac{2}{\bar{m}\left(ac\right)}\right)}^{\frac{1}{2}\bar{m}\left(ac\right)}\cdot \\ {\left(\frac{2}{\bar{m}\left(ac\right)}\right)}^{\frac{1}{2}\bar{m}\left(ac\right)}\cdot {\left(\frac{2}{\bar{m}\left(bc\right)}\right)}^{\frac{1}{2}\bar{m}\left(bc\right)}\cdot {\left(\frac{2}{\bar{m}\left(bc\right)}\right)}^{\frac{1}{2}\bar{m}\left(bc\right)}) \end{array}$$

According to the fact that the geometrical mean is always greater than or equal to the harmonic mean we have:

(57) $$H_{b}\left(\bar{m}\right)\geq {\left(\frac{9}{\sum_{1}^{9}\frac{1}{log{\frac{1}{m(a)}}^{m(a)}}}\right)}^{9}={\left(log{\frac{1}{m(a)}}^{m(a)}\right)}^{9}$$

when m(a):m(b):m(c):m(ab):m(ac):m(bc) = 1:1:1:2:2:2, which means:

(58) $$\min H_{b}\left(\bar{m}\right)={\left(log{\frac{1}{m(a)}}^{m(a)}\right)}^{9}$$

On the other hand $H_{b}\left(m\right)$ can also be denoted as:

(59) $$\begin{array}{c} log_{2}({\left(\frac{1}{m(a)}\right)}^{m(a)}\cdot {\left(\frac{1}{m(b)}\right)}^{m(b)}\cdot {\left(\frac{1}{m(c)}\right)}^{m(c)}\cdot \\ {\left(\frac{2}{m(ab)}\right)}^{\frac{1}{2}m\left(ab\right)}\cdot {\left(\frac{2}{m(ab)}\right)}^{\frac{1}{2}m\left(ab\right)}\cdot {\left(\frac{2}{m(ac)}\right)}^{\frac{1}{2}m\left(ac\right)}\cdot \\ {\left(\frac{2}{m(ac)}\right)}^{\frac{1}{2}m\left(ac\right)}\cdot {\left(\frac{2}{m(bc)}\right)}^{\frac{1}{2}m\left(bc\right)}\cdot {\left(\frac{2}{m(bc)}\right)}^{\frac{1}{2}m\left(bc\right)}) \end{array}$$

According to the fact that the geometrical mean is always less than or equal to the arithmetical mean we have:

(60) $$H_{b}\left(m\right)\leq {\left(\frac{\sum_{i=1}^{9}log{\frac{1}{m(a)}}^{m(a)}}{9}\right)}^{9}={\left(log{\frac{1}{m(a)}}^{m(a)}\right)}^{9}$$

when m(a):m(b):m(c):m(ab):m(ac):m(bc) = 1:1:1:2:2:2, which means:

(61) $$\max H_{b}\left(m\right)={\left(log{\frac{1}{m(a)}}^{m(a)}\right)}^{9}$$

Consequently, the minimum value of $H_{b}\left(\bar{m}\right)$ is ${\left(log{\frac{1}{m(a)}}^{m(a)}\right)}^{9}$, which is exactly the maximum value of $H_{b}\left(m\right)$. Hence $H_{b}\left(\bar{m}\right)-H_{b}\left(m\right)\geq 0$ and thus the following theorem is proved that

(62) $$H_{b}\left(\bar{m}\right)\geq H_{b}\left(m\right)$$

The next proof shows that repeated negation process to a basic probability assignment (BPA) cannot only increase the total uncertainty constantly, but can also converge to the maximum value of the total uncertainty.

**Proof.**  Consider the example above, a frame of discernment Θ={a,b,c}, the BPA vector is denoted as:

(63) $$\vec{m}=\left[\begin{array}{c} \emptyset \\ m(a)\\ m(b)\\ m(c)\\ m(ab)\\ m(ac)\\ m(bc)\\ m(abc) \end{array}\right]$$

According to the proposed negation method, the negation of the BPA can be calculated as

(64) $$\vec{\bar{m}}=E\vec{m}$$

which can be rewritten as

(65) $$\vec{m^{\left(n+1\right)}}=E\vec{m^{\left(n\right)}}$$

where

(66) $$\vec{m^{\left(1\right)}}=E\vec{\bar{m}}$$

(67) $$\vec{m^{\left(0\right)}}=E\vec{m}$$

Thus, $\vec{m^{\left(2\right)}}$ can be denoted as:

(68) $$\vec{m^{\left(2\right)}}=E\vec{m^{\left(1\right)}}=E^{2}\vec{m}$$

Similarly, BPA after repeated negation process is obtained:

(69) $$\lim_{n\to +\infty }\vec{m^{\left(n\right)}}=\lim_{n\to +\infty }E^{n}\vec{m}$$

where *E* is the negation matrix in Equation (43), and $E^{n}$ is obtained as:

(70) $$\lim_{n\to +\infty }E^{n}=\left[\begin{array}{cccccccc} 0 & 0 & 0 & 0 & 0 & 0 & 0 & 0\\ 0 & \frac{1}{9} & \frac{1}{9} & \frac{1}{9} & \frac{1}{9} & \frac{1}{9} & \frac{1}{9} & 0\\ 0 & \frac{1}{9} & \frac{1}{9} & \frac{1}{9} & \frac{1}{9} & \frac{1}{9} & \frac{1}{9} & 0\\ 0 & \frac{1}{9} & \frac{1}{9} & \frac{1}{9} & \frac{1}{9} & \frac{1}{9} & \frac{1}{9} & 0\\ 0 & \frac{2}{9} & \frac{2}{9} & \frac{2}{9} & \frac{2}{9} & \frac{2}{9} & \frac{2}{9} & 0\\ 0 & \frac{2}{9} & \frac{2}{9} & \frac{2}{9} & \frac{2}{9} & \frac{2}{9} & \frac{2}{9} & 0\\ 0 & \frac{2}{9} & \frac{2}{9} & \frac{2}{9} & \frac{2}{9} & \frac{2}{9} & \frac{2}{9} & 0\\ 0 & 0 & 0 & 0 & 0 & 0 & 0 & 1 \end{array}\right]$$

Thus, we get the BPA after repeated negation process:

(71) $$\begin{array}{c} \lim_{n\to +\infty }{\vec{m}}^{n}=\left[\begin{array}{c} m{\left(a\right)}^{\left(n\right)}\\ m{\left(b\right)}^{\left(n\right)}\\ m{\left(c\right)}^{\left(n\right)}\\ m{\left(ab\right)}^{\left(n\right)}\\ m{\left(ac\right)}^{\left(n\right)}\\ m{\left(bc\right)}^{\left(n\right)}\\ m{\left(\theta \right)}^{\left(n\right)} \end{array}\right]=\left[\begin{array}{c} 1/9(1-m(\theta ))\\ 1/9(1-m(\theta ))\\ 1/9(1-m(\theta ))\\ 2/9(1-m(\theta ))\\ 2/9(1-m(\theta ))\\ 2/9(1-m(\theta ))\\ m(\theta ) \end{array}\right] \end{array}$$

□

**Figure 5:**
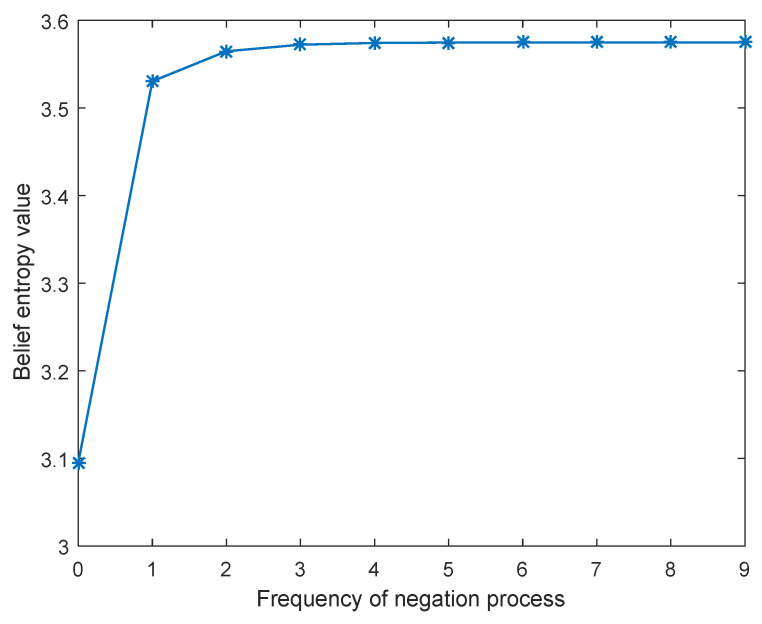
Evolution of total uncertainty as the iteration of negation process increases.

It is noted that $m{\left(a\right)}^{\left(n\right)}$:$m{\left(b\right)}^{\left(n\right)}$:$m{\left(c\right)}^{\left(n\right)}$:$m{\left(ab\right)}^{\left(n\right)}$:$m{\left(ac\right)}^{\left(n\right)}$:$m{\left(bc\right)}^{\left(n\right)}$ = 1:1:1:2:2 for n→+∞ which exactly attains the maximum value of $H_{b}\left(m\right)$ for N=3. The evolution of the total uncertainty is illustrated in Figure 5.

According to the negation of probability proposed by Yager [27], the process of repeated negation can be modeled as a difference equation and the solution of this difference equation for *n* > 2 approaches 1/*n* as i increases. We note also that this corresponds to a maximal entropy allocation of the probabilities shows that the negation of a probability distribution converges to the unique probability distribution, that is each probability value in the probability distribution is 1/*n*, after repeated negation process. Moreover the converged probability distribution exactly attains the most uncertain allocation of the probabilities. Since our negation method is based on the maximal uncertainty, it is necessary of converged BPA after repeated negation process to reach the maximal uncertainty of the system (BPA), since the maximal uncertainty corresponds to a converged BPA. In the discussion part, it is also proved mathematically that the maximal of total uncertainty measure is obtained for m(A) is proportional to |A| which is consistent with the result of the unique converged BPA after repeated negation process.

Consequently, the total uncertainty will indeed increase constantly until the maximum value of the total uncertainty it attained with the increasing iteration of the negation process, which means the proposed negation method is based on the maximum uncertainty.

Compared with the existing negation method of BPA [46], two points are discussed as follows:

1. The existing work tried to present the negation of a mass function the same as the negation of a probability distribution proposed by Yager [27], which means the mass is equally reallocated to other focal elements and the elements in the power set is ignored. However, we believe that the uncertainty of non-singleton elements should be taken into account and the negation of BPA should be extended to the power set. Thus, the proposed negation of a mass function reallocates the corresponding BPA in a weighted manner among the power set.
2. The existing work of negation of a mass function is not based on the maximal uncertainty (entropy). Our work tried to refine this point and reflect the negation of a mass function by total uncertainty measure and proposed a negation method of a mass function based on the maximum total uncertainty mathematically, which is consistent with the negation of a probability distribution based on the maximum entropy proposed by Yager [27].

## 4. Conclusions

In this paper, a novel negation method is proposed to obtain the negation of basic belief assignment in Dempster-Shafer theory. The proposed negation method is implemented in a matrix form to show the reassignment of the BPA intuitively. The proposed negation of BPA reassigns the BPA of a certain element according to the cardinality of intersection of the element and each remaining element in the power set. Particular assumptions have been made for two special elements, the empty set ∅ and the whole set θ in the power set to guarantee that the proposed negation method fits in with our intuition. Closed-world assumption is postulated in this paper to make sure that the frame of discernment is complete and exhaustive, which keeps the BPA of the empty set fixed at 0 no matter how many times the negation process is applied. The BPA of the whole set is assumed to retain unchanged after each negation process is applied since little is known from the whole set regarding where to allocate its BPA, and the whole set dies not reallocate its BPA to the negation. Numerical examples are used to illustrate that how the proposed negation method works for *N* = 1, 2 and 3. Meanwhile, the proposed negation method reduces to the negation of probability distribution as all elements in the power set are singletons. Total uncertainty measures are used to measure the uncertainty in this paper due to the proposed negation method acting in a manner that increases the total uncertainty measure. It is found that the proposed negation method converges to a certain BPA distribution after repeated negation process, which exactly attains the maximum value of the total uncertainty measure. This also shows that our proposed negation method is based on the maximum uncertainty. Therefore, not only does this paper extend the negation of probability to BPA, but it also preserves the properties of the negation of probability.

## Figures and Tables

**Table 1:** BPA value for each element and the total uncertainty corresponding to each negation process.

Frequency of Iterations	m(a)	m(b)	m(c)	m(ab)	m(ac)	m(bc)	m(abc)	Total Uncertainty
0	0.1000	0.1500	0.0000	0.0000	0.3000	0.2500	0.2000	3.0952
1	0.1083	0.1167	0.0417	0.2250	0.1500	0.1583	0.2000	3.5305
2	0.0792	0.0750	0.1125	0.1542	0.1917	0.1875	0.2000	3.5647
3	0.0937	0.0958	0.0771	0.1896	0.1708	0.1729	0.2000	3.5723
4	0.0865	0.0854	0.0948	0.1719	0.1812	0.1802	0.2000	3.5742
5	0.0901	0.0906	0.0859	0.1807	0.1760	0.1766	0.2000	3.5747
6	0.0883	0.0880	0.0904	0.1763	0.1786	0.1784	0.2000	3.5748
7	0.0892	0.0893	0.0882	0.1785	0.1773	0.1775	0.2000	3.5749
8	0.0887	0.0887	0.0893	0.1774	0.1780	0.1779	0.2000	3.5749
9	0.0890	0.0890	0.0887	0.1780	0.1777	0.1777	0.2000	3.5749
10	0.0889	0.0888	0.0890	0.1777	0.1778	0.1778	0.2000	3.5749
11	0.0889	0.0889	0.0888	0.1778	0.1778	0.1778	0.2000	3.5749
12	0.0889	0.0889	0.0889	0.1778	0.1778	0.1778	0.2000	3.5749
13	0.0889	0.0889	0.0889	0.1778	0.1778	0.1778	0.2000	3.5749
14	0.0889	0.0889	0.0889	0.1778	0.1778	0.1778	0.2000	3.5749
15	0.0889	0.0889	0.0889	0.1778	0.1778	0.1778	0.2000	3.5749

**Table 2:** Value of uncertainty measured by different measures.

Uncertainty Measures	0	1	2	3	4	5	6	7
$H_{b}\left(m\right)$	3.5084	3.5726	3.5743	3.5747	3.5748	3.5749	3.5749	3.5749
$H_{rp}\left(m\right)$	2.5511	2.4349	2.4352	2.4352	2.4353	2.4353	2.4353	2.4353
$H_{D}\left(m\right)$	4.1331	4.1291	4.1308	4.1312	4.1312	4.1313	4.1313	4.1313

**Table 3:** Distribution of BPA after negation.

$\bar{m}\left(a\right)$	$\frac{1}{6}\left(b+c+2bc\right)$
$\bar{m}\left(b\right)$	$\frac{1}{6}\left(a+c+2ac\right)$
$\bar{m}\left(c\right)$	$\frac{1}{6}\left(a+b+2ab\right)$
$\bar{m}\left(ab\right)$	$\frac{1}{6}\left[a+b+2\left(c+ac+bc\right)\right]$
$\bar{m}\left(ac\right)$	$\frac{1}{6}\left[a+c+2\left(b+ab+bc\right)\right]$
$\bar{m}\left(bc\right)$	$\frac{1}{6}\left[b+c+2\left(a+ab+ac\right)\right]$
